# 

**Published:** 1915-09

**Authors:** 


### CONTENTS.

ORIGINAL COMMUNICATIONS—

The Blood,—By Dr. Charles W. Gould, M. D. . . . . . 261

Partial Paralysis of the Soft Palate Following Removal of Tonsils and Adenoids.. Observations on the Tonsil and Adenoid Operation—By Dunbar Roy, M. D., Atlanta, Ga. . . . . . . . . . . . . . . . . . . . . . . 266

Some Observations on the Use and Abuse of Opium—By M. T. Davis, M. D., Atlanta, Ga. . . . . . . . . . . . . . 276

Addenda to Paper on Opium Habit . . . . . . . . . . . . . . . . 280

Venarsen in Tertiary Syphilis—By M. T. Davis, M. D., Atlanta, Ga. . . . . . . . . . . . . . . . . . . . . . . . . . . . 281

Surgical Suggestions . . . . . . . . . . . . . . . . . . . . . . . . . . . . 282

Regular Meeting of Fulton County Medical Society, October 7, 1915.—By Dr. Allen H. Bunce. . . . . . 283

EDITORIAL . . . . . . . . . . . . . . . . . . . . . . . . . . . . . . . . . . 286

